# Supplementary material for: Causal Relationship between Aging and Anorexia Nervosa: A White-Matter-Microstructure-Mediated Mendelian Randomization Analysis
Source: Biomedicines. 2024 Aug 16;12(8):1874. doi: 10.3390/biomedicines12081874 (PMC11351342; doi:10.3390/biomedicines12081874)
Supplement: Supplementary file 1 [file biomedicines-12-01874-s001.zip › Supplementary Figure/Figure S9/Leaveoneout plot of MR analysis for ubm-b-1960 to AN.pdf]

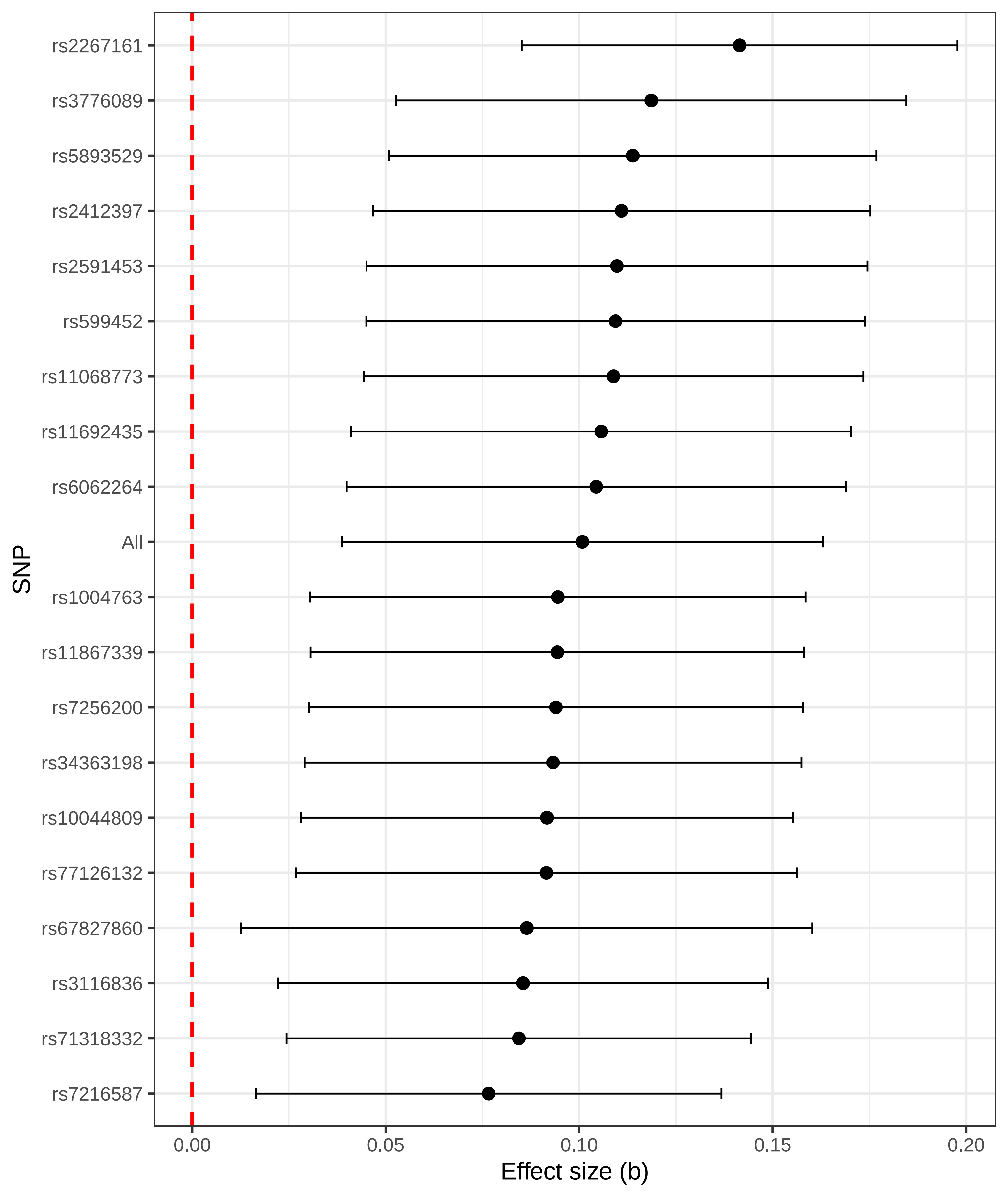

rs2267161
rs3776089
rs5893529
rs2412397
rs2591453
rs599452
rs11068773
rs11692435
rs6062264
All
rs1004763
rs11867339
rs7256200
rs34363198
rs10044809
rs77126132
rs67827860
rs3116836
rs71318332
rs7216587
SNP
0.00
0.05
0.10
0.15
0.20
Effect size (b)
